# Clinical features of dementia with lewy bodies in 35 Chinese patients

**DOI:** 10.1186/2047-9158-3-1

**Published:** 2014-01-08

**Authors:** Ding Han, Qiong Wang, Zhongbao Gao, Tong Chen, Zhenfu Wang

**Affiliations:** 1 Department of Neurology, South Building, General Hospital of Chinese PLA, Beijing 100853, China

**Keywords:** Dementia with Lewy bodies, Secondary literature evaluation, Clinical features, Neurodegenerative disease

## Abstract

**Objective:**

To investigate the clinical features of dementia with Lewy bodies (DLB) in a Chinese population.

**Methods:**

Computer-based online searches through China Biology Medicine disc and China National Knowledge Infrastructure were performed to collect case reports of DLB published between 1980 and 2012. Clinical characteristics were analyzed.

**Results:**

A total of 18 studies comprising 35 patients (26 males and 9 females) were included. The mean age at onset was 67.2 ± 9.8 years. Onset was characterized by memory impairment and accounted for 58.8% of all cases, followed by parkinsonism (11.8%), visual hallucinations (8.8%), and compulsive personality disorder (2.9%). The other patients (17.6%) presented two of the three core features of DLB at onset. With disease progression, parkinsonism was reported in 100% of cases, followed by visual hallucinations (97.1%), psychiatric symptoms (85.7%), severe neuroleptic sensitivity (81.8%), fluctuating cognition (68.6%), repeated falls (40.0%), sleep disorders (22.9%), and transient loss of consciousness (17.1%). 26 patients who were subjected to Mini-Mental State Examination scored ≤ 24. 10 patients presented relative preservation of hippocampus and medial temporal lobe structures on CT/MRI scan. Occipital hypometabolism occurred in 2 of 3 patients who underwent SPECT/PET perfusion scan. 12 patients showed an increasing of slow frequency activity on EEG, prominently in frontal and temporal lobes.

**Conclusions:**

DLB often strikes elderly individuals. Its clinical core features are dementia, fluctuating cognition, recurrent visual hallucinations and spontaneous features of parkinsonism. Neuropsychological, neuroimaging and EEG examinations may improve the diagnostic accuracy and discriminate DLB from other dementias.

## Introduction

Dementia with lewy bodies (DLB) is the second most prevalent cause of degenerative dementia after Alzheimer disease (AD) in older people, accounting for 10% of all cases [1]. Its clinical features include dementia, fluctuating cognition, visual hallucinations and parkinsonism [2]. The main neuropathological findings are Lewy bodies (LBs), senile plaques (SP) and less or no neurofibrillary tangles (NFT) [3]. To date, DLB is more common than previously thought. However, less systematically studies of DLB are published in China. The present study aimed to review clinical features of DLB through a thorough analysis of Chinese DLB reports published from 1980 to 2012.

## Data and methods

### Subjects

#### Data inclusion and exclusion criteria

Pre-indexing did not reveal any systematic evaluations, prospective cohort studies, retrospective cohort studies, bioecological studies, or non-consecutive cohort studies of DLB in China. Case or individual reports published between January 1, 1980 and November 1, 2012 were included, but reviews and experimental studies of DLB were excluded.

#### Subject inclusion criteria

The current criteria for DLB diagnosis in China have been defined by the DLB Consortium in 2005. Dementia is the essential condition for a diagnosis of possible or probable DLB. The core features of DLB include fluctuating cognition with pronounced variations in attention and alertness, recurrent visual hallucinations that are typically well formed and detailed, and spontaneous features of parkinsonism. Two or more core features is sufficient for a diagnosis of probable DLB, one for possible DLB. The suggestive features of DLB include rapid eye movement sleep behavior disorder (RBD), severe neuroleptic sensitivity, and low dopamine transporter uptake in basal ganglia demonstrated by SPECT or PET imaging. If one or more of suggestive features is present in the presence of one or more core features, a diagnosis of probable DLB can be made. In the absence of any core features, one or more of suggestive features is sufficient for possible DLB. Probable DLB should not be diagnosed on the basis of suggestive features alone [2].

#### Retrieval methods

“Dementia with lewy bodies, DLB, lewy body dementia, diffuse lewy body disease, DLBD, lewy body disease” were used as key words to search the China Biology Medicine disc and China National Knowledge Infrastructure for related articles published between 1980 and 2012.

#### Data extraction

Age at onset, initial symptoms, clinical manifestations, laboratory examinations were extracted and recorded for each patient. The data were analyzed by SPSS version 13.0.

## Results

A total of 81 articles related to DLB were collected. 63 articles were excluded for duplication, non-clinical research or lacking of essential clinical materials. A total of 18 studies comprising 35 DLB patients were included (26 males and 9 females), with a mean onset age of 67.2 ± 9.8 years (range 40–81 years). General characteristics of the included articles are listed in Table 1. There were no significant differences between male and female patients in terms of age of onset (male: 67.8 ± 8.0 years, female: 65.5 ± 15.2 years, *P* > 0.05).

**Table 1:** General characteristic of included cases

***Article***	***Case***	***Gender***	***Age of onset***
	***number***	**(*male/female *)**	**(*male/female*)**
Lu H et al. [4]	1	1/0	66/
Wang Y et al. [5]	1	0/1	/77
Gai QW et al. [6]	2	2/0	69,67/
Yang GE et al. [7]	1	1/0	50/
Chen L et al. [8]	8	6/2	63 ~ 72
Zhang XQ et al. [9]	1	0/1	/63
Wu XP [10]	4	2/2	68,66/58, unknown
Yang WM [11]	3	3/0	56,56,75/
Li YM et al. [12]	1	1/0	70/
Wang EZ [13]	1	0/1	/40
Xian HT [14]	2	2/0	68,75/
W R et al. [15]	1	0/1	/74
Li JL et al. [16]	1	1/0	72/
Liu XS et al. [17]	4	4/0	71,74,76,77/
Wang XH et al. [18]	1	1/0	53/
Fu YH et al. [19]	1	0/1	/81
Yang L et al. [20]	1	1/0	70/
Lu WF et al. [21]	1	1/0	76/

### Clinical manifestations

Of the 34 patients who had detailed initial clinical manifestation records (1 patient’s initial clinical manifestation was not mentioned), 20 patients (58.8%) showed cognitive decline, 4 (11.8%) showed parkinsonism, 3 (8.8%) showed visual hallucinations, 3 (8.8%) presented cognitive decline with parkinsonism, 2 (5.9%) presented cognitive decline with visual hallucinations, 1 (2.9%) presented visual hallucinations with parkinsonism.

During the progression of the disease, 24 patients (68.6%) displayed fluctuating cognition that appeared within one day to several weeks. 34 patients (97.1%) suffered from recurrent visual hallucinations that were well formed and detailed. 35 patients (100%) manifested the symptoms of parkinsonism, including rigidity, bradykinesia, postural instability and gait difficulty (8 patients presented rest tremor). 8 patients (22.9%) had sleep disorder, 4 of them exhibited RBD. 14 patients (40%) had the experience of repeated falls because of postural instability (12 patients), transient loss of consciousness (1 patient) and paroxysmal vertigo (1 patient). 1 patient (2.9%) presented orthostatic hypotension. 30 patients (85.7%) had neuropsychiatric symptoms, including irritability, apathy, auditory hallucination, illusion, anxiety and depression.

3 patients were confirmed by autopsy. The disease courses of the 3 patients were 2.5 years, 3 years and 11 years respectively. The initial symptoms were parkinsonism with hallucinations, parkinsonism with cognitive decline, and cognitive decline respectively. During the progression of the disease, all the 3 patients shared the same clinical features of parkinsoniam and cognitive decline. 2 of them presented visual hallucinations. 2 patients had exacerbated symptoms after the treatment of anti-parkinsonism drugs. 2 patients were died from multiple organ failure and 1 was from stroke. All the 3 patients showed the similar pathological results: diffuse brain atrophy dominantly in medial hippocampus, parahippocampal gyrus, entorhinal cortex and amygdala. LBs were widely distributed in the cortical and subcortical regions of the brain, including substantia nigra, locus coeruleus, frontal lobe, parietal lobe, cingulate gyrus, meynert’s nucleus, et al. Further pathological features were not mentioned.

### Laboratory examinations

A total of 26 patients were subjected to Mini-Mental State Examination (MMSE) evaluation and mean score was (12.2 ± 5.6). 34 patients who underwent CT/MRI examinations presented diffuse brain atrophy, 10 of them (29.4%) showed relative preservation of medial temporal lobe and hippocampus, and 1 patient (2.9%) showed prominent temporal lobe atrophy. Of the 3 patients who underwent SPECT/PET examinations, 1 patient showed low dopamine transporter uptake in right striatum, 1 showed low fluorodeoxyglucose uptake in left frontal lobe, bilateral posterior temporal, parietal and occipital lobes, and the last one showed generalized low uptake. Of the 19 patients who underwent electroencephalogram examinations, 12 patients showed an increasing of slow frequency activity in frontal and temporal lobes, 4 showed mild to moderate abnormalities, and 3 showed normal results. 1 patient underwent polysomnography examination and the result was abnormal.

### Therapy

Of the 24 patients who were treated with L-dopar, 12 patients had a mild to moderate improvement in extrapyramidal symptoms, 11 patients had no significant improvement, 1 patient was subject to exacerbation. 2 patients who were treated with selegiline had a mild improvement. Of the 5 patients who had used artane, 2 patients had no significant improvement, and 3 patients suffered from aggravating hallucinations and psychiatric symptoms. 2 patients who were treated with amantadine had no significant improvement. 1 patient was reported to have a bad performance after taking piribedil. Of the 12 patients who had used the neuroleptic agents, 11 patients showed severe neuroleptic sensitivity (6 patients used perphenazine, 2 patients used risperidone, 1 patient used clozapine, 1 patient used olanzapine, 1 patient used haloperidol), and 1 patient had a mild improvement after using a small dose of quetiapine. 8 patients who were treated with donepezil (5-10 mg/d) had a mild to moderate improvement in dementia and neuropsychiatric symptoms.

## Discussion

DLB is a neurodegenerative disease. It is reported a prevalence range from 0 to 5% with regard to the general population, and the incidence of DLB is 0.1% a year for the general population [22]. An accumulation of proteins including α-synuclein aggregates in cortical and subcortical regions of the brain is the main neuropathological findings [23]. Its core clinical features include fluctuating cognition with pronounced variations in attention and alertness, recurrent visual hallucinations that are typically well formed and detailed, and spontaneous features of parkinsonism [2]. Despite its high prevalence, few cases have been reported in China, and no related epidemiological data has been reported too. We aim to systematically investigate the clinical features of DLB in a Chinese population as well as to raise the clinicians’ consciousness of the disease through this article.

In the present study, the mean age of onset was (67.2 ± 9.8) years (range from 40 ~ 81years), which was consistent with previous reports [24]. There were no significant differences between males and females in age of onset (*P* > 0.05). The present study confirmed that cognitive decline was the most commonly reported symptoms at disease onset, whereas visual hallucinations and parkinsonism were less likely to happen. Previous studies found deficits on tests of attention, executive function, and visuospatial ability may be especially prominent in the early stage and memory impairment may not necessarily occur [2], which was not consistent with the present result of memory loss being much more common at disease onset. Most patients in the present study showed bilateral symmetric parkinsonism with prominent axial rigidity and less rest tremor. Half of 8 cases who had sleep disorders presented RBD, the incidence rate of which was lower than it was previously reported [25]. Ferman et al. [26] have found that inclusion of RBD as a core clinical feature improves the diagnostic accuracy of autopsy-confirmed DLB. When RBD was added and clinically probable DLB reflected 2 or more of 4 features, sensitivity improved to 88%. When dementia and RBD were also designated as probable DLB, sensitivity increased to 90% while specificity remained at 73%. A high proportion of patients with neuropsychiatric symptoms were found in this study. Neuropsychiatric symptoms may complicate the clinical diagnosis of DLB [27], patients who presented neuropsychiatric symptoms at onset were often misdiagnosed for psychosis. In the present study, 30 patients showed dementia occurred before parkinsoniam, and 1 case showed dementia occurred after paikinsoniam within half a year. The other 4 patients presented dementia occurred 2–3 years later in the context of confirmed paikinsonism. These 4 patients fulfilled the clinical criterion of probable DLB. The existing 1-year rule suggests Paikinson disease dementia (PDD) should be used to describe dementia that occurs at least 1 year later than parkinsonism. However, Tsuboi et al. [28] found that PDD and DLB shared few differences at autopsy, so 1 year rule may be not compatible for all DLB patients. Since no pathological findings of the 4 patients have been reported, we couldn’t confirm that they share the same features with Tsuboi’s cases. 29.4% of patients who underwent CT/MRI examinations showed relative preservation of medial temporal lobe and hippocampus, which is helpful to distinguish from AD patients who presented marked atrophy in temporal lobe and hippocampus [29]. 2 patient who underwent FDG-PET scan displayed low metabolism in bilateral occipital lobes, which is consistent with the result of Feng’s [30] and is helpful for differentiating DLB from other types of dementia. Another patient exhibited low dopamine transporter uptake in right striatum, which had an important significance for differentiation between DLB and AD. However, the utilization of SPECT/PET has its own limitation on differentiating from PD [31-33]. 3 patients who were confirmed by autopsy presented diffused LBs in cortical and subcortical regions of the brain, which was consistent with McKeith’s study [34].

To date, the evidence base for making recommendations about the management of DLB is limited and what follows is based upon consensus opinion of clinicians experienced in treating DLB. Supportive and symptomatic therapies are still the main approaches. The observation of less neuronal loss in DLB than in AD suggests that cortical neurons in DLB are more viable than those in AD and could be more responsive to cholinergic stimulation [35,36]. Cholinesterase inhibitors (ChEIs) may be of benefit for the fluctuating cognitive impairments with impact on global function and activities of daily living [37]. It is reported that ChEIs, including rivastigmine, galantamine and donepezil, may improve patients in measures of attention as well as reduce in psychotic symptoms [38-41]. In the present study, a total of 8 patients who have been treated with donepezil have varying degrees of improvement in dementia and neuropsychiatric symptoms, which is consistent with previous studies. However, we have no data about rivastigmine and galantamine in DLB therapy among our collected patients.

Molloy et al. [42] found that L-dopa is generally well tolerated in DLB but produced a significant motor response in only about one third of patients. Bonelli et al. [43] found a greater than 20% improvement in UPDRS scores in 50% of DLB patients compared to 65% of Parkinson’s disease with dementia patients and 90% of Parkinson’s disease patients. Goldman et al. [44] reported improvement in motor symptoms in 6 of 19 DLB patients. Of the responders, only 22% of the cohort is able to achieve improvement in motor symptoms without exacerbation of psychosis. In our study, 12 patients (50%) who have taken L-dopa have a mild to moderate improvement in extrapyramidal symptoms, and 1 patient (4%) has a psychosis exacerbation. The UPDRS scores of these patients are not reported. The effectiveness of motor benefit is consistent with previous reports. However, the risk of psychosis exacerbation is lower. Although the effectiveness of L-dopa for DLB is lower than PD, it may be beneficial to the motor symptoms in the long run [42,45]. 2 patients who are treated with selegiline have been reported an improvement in extrapyramidal symptoms, which suggest that monoamine oxidase B (MAOB) may have applied value in DLB therapy.

30 patients have suffered from a variety of neuropsychiatric symptoms. 11 patients who have taken neuroleptic agents have presented severe neuroleptic sensitivity. It is suggested that typical antipsychotics (including perphenazine, haloperidol, et al.) should be avoided [46]. Of the 5 patients who have taken atypical antipsychotics, only 1 patient has a mild improvement after taking quetiapine. Thus ChEIs may be the first option [38,40]. However, if ChEIs are ineffective or if more acute symptom control of behavior is required, it may be difficult to avoid a cautious trial of an atypical antipsychotic [2].

In conclusion, Chinese DLB patients usually begin with the symptoms of cognitive impairment which is characterized by memory loss. With disease progression, most patients present at least 2 or 3 core clinical features of DLB including the symptoms of parkinsonism, visual hallucinations and fluctuating cognition. The occurrence of RBD may improve the diagnostic accuracy of DLB. Patients often suffer from psychotic symptoms which may be exacerbated by using neuroleptic agents and some anti-parkinsonism drugs. ChEIs may be the first option to improve the cognition and psychotic symptoms. L-dopar may have a limited improvement in parkinsonism symptoms. MAOB may be effective. Occipital low uptake on FDG-PET may have significance in differentiating DLB from other dementia. Other laboratory examinations may improve the diagnostic accuracy. However, there are some problems as followed. First, few pathological findings of Chinese DLB have been reported during the past 30 years, which may influence our further understanding of the disease. Second, DLB have overlapped symptoms with AD, PD, PDD, psychosis and other diseases, which may confuse clinicians to make misdiagnosis. Third, not all the patients in our study have detailed clinical manifestations or medications, our analyses and discussions are limited. Therefore, detailed physical and laboratory examinations, reasonable therapies, and long time follow-up are very important. Early and correct diagnosis and medical treatment provide better control for clinical symptoms in DLB, as well as improve quality of life. The present study reviewed literature from China for the past 30 years and analyzed clinical data from 35 cases. Results provided a primary reference for better understanding Chinese DLB features and could help to improve the early diagnostic rates, however, more researches are needed for the limitation of few cases.

## Competing interests

The authors declare that they have no competing interests.

## Authors’ contributions

DH and QW designed the study and wrote the manuscript. ZW was responsible for manuscript revision. ZG and TC were responsible for data integration, analysis and statistical analyses. All authors read and approved the final manuscript.
